# Development of the RIPPLES framework for patient and public involvement in rapid evidence syntheses

**DOI:** 10.1186/s40900-026-00878-5

**Published:** 2026-04-15

**Authors:** Eugenie Evelynne Johnson, Sean Gill, Madeleine Still, Daisy Trenchard, Debbie Smith, Rebecca Harmston, Jane McDermott, Fiona Pearson

**Affiliations:** 1 NIHR Innovation Observatory, Newcastle University, Newcastle upon Tyne, UK; 2 Population Health Sciences Institute, Newcastle University, Newcastle upon Tyne, UK; 3 Public Research Team Member, Newcastle upon Tyne, UK

**Keywords:** Patient involvement, Patient engagement, Public involvement and engagement, Framework, Evidence synthesis, Rapid evidence synthesis

## Abstract

**Background:**

Patient and public involvement (PPI) is an expectation and requirement of health and social care research within the United Kingdom. Although there is available guidance on how to practice PPI, to date there have been no frameworks or resources available specifically for PPI in rapid evidence syntheses, where work can be completed in as little as two or three weeks. Our objective was to design a framework to support those doing rapid evidence syntheses to include PPI in their work.

**Methods:**

We developed the framework across three stages. First, we undertook a scoping review of published PPI frameworks in health and social care (*N* = 53) and a survey of key interest-holders (*N* = 101). The results of these alongside an online workshop with 15 interest-holders (researchers, members of the public, policymakers, PPI professionals) in November 2024 informed the initial development of the framework. We evaluated the draft framework at a second online workshop with 16 interest-holders in January 2025. We analysed the findings from this second workshop to further refine the framework.

**Results:**

The Rapid Involvement of Patients and the PubLic in Evidence Synthesis (RIPPLES) framework guides PPI in rapid evidence syntheses through three interconnected layers: core guiding principles of the UK Standards for Public Involvement at the centre; building continued approaches to patient, public and community engagement; and steps for embedding PPI into individual rapid evidence synthesis projects. An accompanying guidance document and worksheet to plan PPI activities were also developed to aid researchers in using the framework.

**Conclusions:**

RIPPLES is the first framework to provide practical and pragmatic guidance on how to embed PPI within rapid evidence synthesis. The framework will undergo evaluation and refinement, including development of public-facing materials and case studies to supplement current resources.

**Supplementary Information:**

The online version contains supplementary material available at 10.1186/s40900-026-00878-5.

## Plain language summary

Rapid evidence syntheses are a type of research that brings together all the information about a specific topic to find out what it says overall. They sometimes need to be done in just a couple of weeks. We know involving patients and members of the public in evidence syntheses has benefits for the people involved and the research itself. However, researchers have not had guidance on how to involve people in rapid evidence syntheses. This is why we developed the Rapid Involvement of Patients and the PubLic in Rapid Evidence Synthesis (RIPPLES) framework. We developed RIPPLES by finding common features of already-published frameworks for patient and public involvement, doing a survey and holding two workshops with members of the public, researchers who do evidence syntheses and people who work in public involvement. We brought all three of these pieces of work together to co-develop RIPPLES. RIPPLES builds on the UK Standards for Public Involvement. It encourages researchers to develop relationships with members of the public and communities so they can start involvement in rapid evidence syntheses quickly. Finally, it gives researchers guidance on how to involve people in rapid evidence synthesis projects. The framework comes with a detailed guidance document with links to other useful information and a worksheet to help plan patient and public involvement in rapid evidence syntheses. RIPPLES will help researchers meaningfully involve patients and the public in rapid evidence syntheses. We will also evaluate it in future to make sure it continues to be useful.

**Supplementary Information:**

The online version contains supplementary material available at 10.1186/s40900-026-00878-5.

## Background

Evidence synthesis is a research method that combines information from multiple studies looking at the same topic to understand their findings as a whole [1]. In the UK, the National Institute of Health and Care Research (NIHR) expects patient and public involvement (PPI) to be embedded within all research that it funds. The benefits of PPI in evidence syntheses to both the public and researchers have been documented. They include, but are not limited to: greater empowerment and knowledge for the public; enhancing the relevance of research to end users; and enhancing how findings are shared with others [2]. 

Specific tools to help researchers embed PPI into evidence syntheses already exist, including the Authors and Consumers Together Impacting on eVidencE (ACTIVE) framework [3], while the NIHR also provides extensive guidance on best practice for PPI, including the UK Standards for Public Involvement and briefing notes for researchers [4, 5]. Despite this, there remain challenges in how PPI is conducted and reported in evidence syntheses. A recent cross-sectional analysis of papers published in a single journal in 2020 reported that only 12.5% of 376 systematic reviews and 8.9% of 265 meta-analyses embedded any form of PPI [6].

This issue is compounded in the case of rapid evidence syntheses. Rapid evidence syntheses, sometimes called rapid reviews, follow a streamlined process to produce summaries of evidence in a timely manner [7]. The duration of these reviews can vary, but is sometimes as little as a few weeks [7]. Although the importance of embedding the perspectives of knowledge users into rapid evidence syntheses has been stressed [8], the challenge of embedding PPI into this form of research appears even greater. In a further cross-sectional study, only 6 of 103 rapid reviews sampled involved patient partners in their process [9]. 

The need for defined guidance on how to embed meaningful PPI into rapid evidence syntheses was emphasised by PRIORITY III, which used a priority setting partnership based on the methods of the James Lind Alliance to identify research priorities on how to plan, undertake and share the results of rapid reviews in a healthcare context. PRIORITY III found that identifying ways to meaningfully involve interest-holders, including PPI, within these projects was the foremost priority methods topic for rapid evidence syntheses [10]. However, to date, there has been no defined, specific guidance or frameworks to aid researchers wishing to embed PPI into rapid evidence syntheses.

As such, we developed the Rapid Involvement of Patients and the PubLic in Rapid Evidence Synthesis (RIPPLES) framework for PPI in rapid evidence synthesis to address this knowledge gap. Here, we explain its development, components and potential future directions.

## Methods

**Fig. 1:**
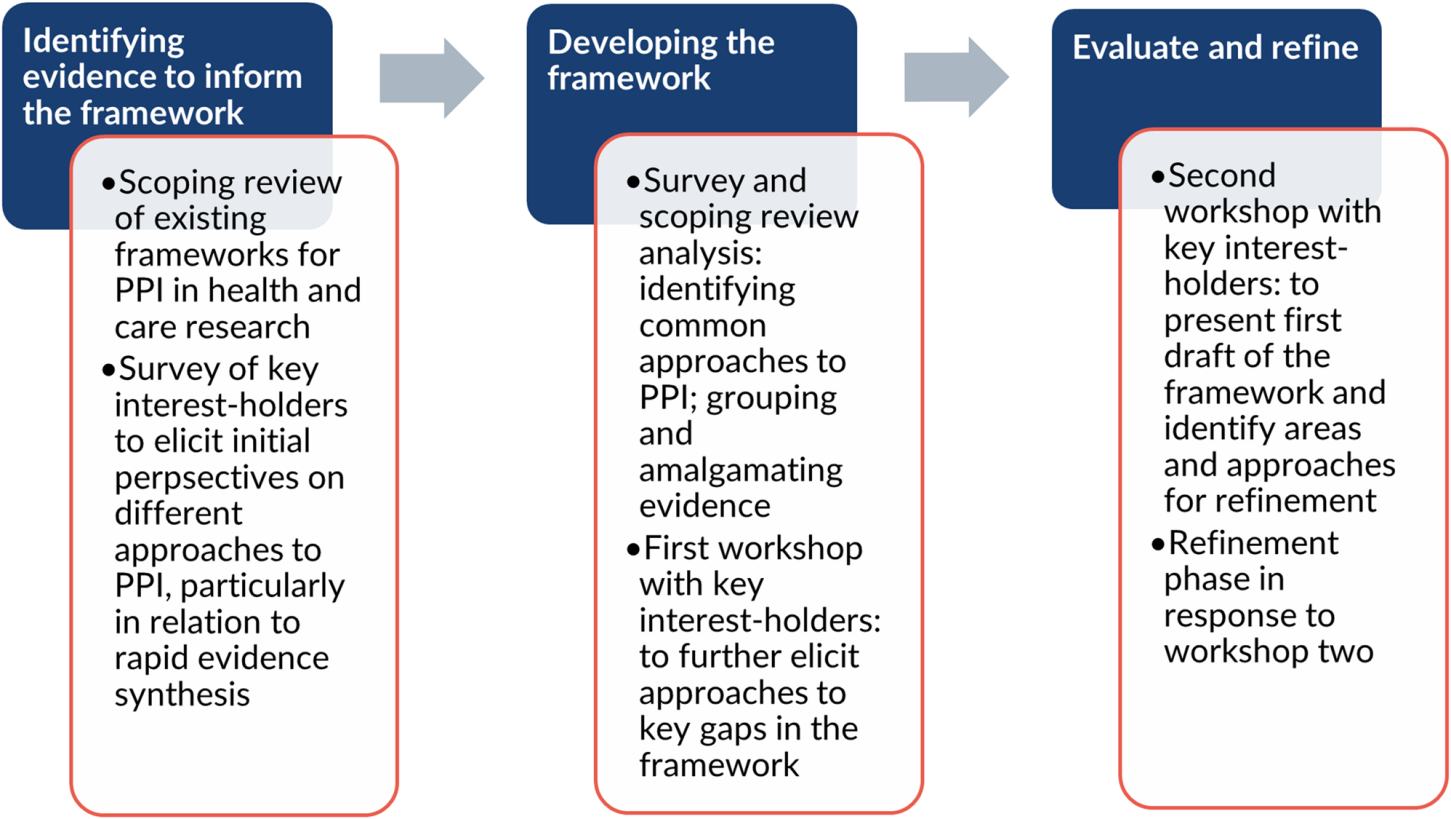
Process to develop the RIPPLES framework

To develop the RIPPLES framework, we followed a three-stage process using adapted suggestions provided by McMeekin et al. (2020); [11] see Fig. 1. We detail the methods at each stage in the following sections. Ethical approval for the survey and workshops was obtained by Newcastle University on 25 July 2024 (reference: 2796/48332).

### Patient and public involvement

**Fig. 2:**
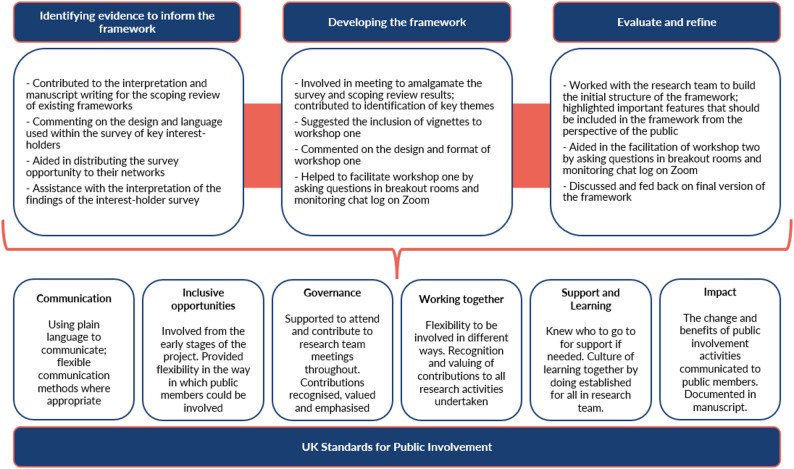
Overview of public involvement in the development of RIPPLES

We involved members of the public, including two public partners (DS, RH) from the beginning of the project and throughout the development of the framework. Both DS and RH are members of the NIHR Innovation Observatory’s Public Advisory Group and have experience in being involved in health and social care research; both also brought their lived experience of health and social care. However, both were less experienced specifically in rapid evidence synthesis methodologies. An overview of how both DS and RH were involved in each stage of the development process is summarised in Fig. 2. DS and RH also contributed to the reviewing of this manuscript. Both public partners were recognised for their time at NIHR recommended rates [12]. The GRIPP-2 short form checklist can be found in Appendix 1 [13]. 

### Stage 1: Identifying evidence to inform the framework

We used two methods to gather evidence to inform the draft framework. The first was a scoping review of published PPI frameworks in health and social care. The methods and results of this scoping review have been reported in detail elsewhere [14]. 

Furthermore, we conducted an online survey using Qualtrics [15]. As well as an overview of the project and information on confidentiality and how any personal data would be used, the survey asked multiple-choice questions regarding their perspectives on how PPI in rapid evidence synthesis could be implemented. These questions were based around concepts used within the ACTIVE framework and the UK Standards for Public Involvement [3, 4]. Two items from the ACTIVE framework relating to how public partners are identified to be involved in reviews and how they are interacted with were adapted into plain language multiple-choice questions [3]. These questions asked respondents to consider how people could be identified to be involved in a rapid evidence synthesis and how they would best be interacted with. The six UK Standards for Public Involvement were also explained in plain language and respondents were asked to identify which would be considered important when involving people within rapid evidence syntheses [4]. All phrasing used was reviewed by our public partners (DS and RH) and the survey was piloted internally within the research team. The final survey can be found in Appendix 2. We distributed the survey to a list of organisations and networks with an interest in PPI or evidence synthesis, or both, in August 2024 and employed snowball sampling, whereby those we contacted were encouraged to cascade the survey to anyone with a potential interest in the area. The survey remained open for two weeks.

Once closed, we collated responses in Microsoft Excel where, prior to analysis, we removed responses that did not answer any of the questions relating to PPI in rapid evidence synthesis or from those under 18 years old. One researcher (DT) used Stata 18 to analyse the multiple-choice questions using frequencies and percentages. Open-ended questions were coded inductively by one researcher (EEJ) and checked by a second (SG).

Common findings from the scoping review and survey were merged, with key concepts and challenges summarised and stratified into smaller sub-concepts. Members of the research team (EEJ, SG, MS, DT, DS, RH) identified important concepts for inclusion in the framework, as well as areas that needed further development at Stage 2.

### Stage 2: Developing the framework

We held two workshops to develop the framework based on the findings from Stage 1. The first workshop in November 2024 aimed to gather insights surrounding different approaches to PPI in rapid evidence synthesis that remained unclear from Stage 1. In the second workshop, held in January 2025, we presented an initial draft of the framework and discussed potential omissions, areas for clarification and potential implementation challenges and facilitators.

To recruit for both workshops, we had included an expression of interest form at the end of the interest-holder survey and sent further emails to networks and organisations with an interest in the topic area. A purposive sampling approach was used to select members of the public, researchers and other interest-holders to participate. An information sheet and consent form were developed within Qualtrics and sent to participants (see Appendix 3).

Both workshops were held online via Zoom and included: general housekeeping; a background to the project and the aims of the session prior to activities; whole-group Zoom polls; and two breakout discussion sessions (see Appendix 4 for full agendas). In response to our public research team members’ suggestions, for workshop one we created a short vignette to help elicit responses (see Appendix 5). Both workshops, including breakout rooms, were facilitated by members of the research team (EEJ, SG, MS, DT, DS, RH, JM), with technical support from colleagues within the NIHR Innovation Observatory.

Following workshop one, notes were gathered by all facilitators, anonymised and coded against a framework within Microsoft Excel. The final coding frameworks are presented in Appendix 6. Data from each breakout room were coded by pairs of researchers (of EEJ, SG, MS and DT), with one conducting initial coding and another checking for accuracy. Any discrepancies between reviewers were discussed; had discrepancies not been resolved, another reviewer would have arbitrated the decision. Members of the research team (EEJ, SG, MS, DT, DS, RH and JM) then took the results of the scoping review, survey and workshop to create a draft framework structure during a two-hour online session. We used Padlet to organise and group features collectively. Finally, two researchers (EEJ and MS) took the results of the Padlet exercise and created draft visualisations for the framework. These were discussed with other research team members, with visual features merged as appropriate to create the first draft framework (see Appendix 7).

### Stage 3: Evaluating and refining the framework

We took the initial draft of the framework to the second online workshop (see Appendix 8 for the agenda). We presented the draft framework and explained its principles in brief, using breakout rooms to discuss the structure, content and its potential implementation in more depth.

All breakout discussions were recorded and transcribed using Zoom before being checked for accuracy and anonymised by a single researcher (EEJ) prior to analysis. The analysis process was the same as workshop one. Following refinement of the framework and its components, four researchers (EEJ, DT, SG, MS) worked to draft accompanying guidance for the framework, including pragmatic approaches to each concept and links to useful relevant sources of further information.

## Results

### Results of the scoping review, survey and workshops

Results of the scoping review have been detailed in a prior publication [14]. In total, 101 responses to the survey were analysed. Of those who participated, 47 (47%) were members of the public, 28 (28%) were researchers, two (2%) were clinicians and to (2%) were policymakers. The remaining 21 (21%) participants chose to describe themselves as “other,” including PPI professionals (such as people whose main role is to support researchers in embedding PPI into their own work and more broadly across institutions). Most of the respondents were of White ethnicity (61%) and 71 (71%) were living in the UK. Almost half (49.5%) had not been involved in a rapid evidence synthesis previously. Thirty-three respondents (35.48%) believed that having an open call for people to be involved and taking a flexible approach to who is involved throughout was the best approach to finding people to be involved in rapid syntheses, with the same number suggesting that having an open call for people to be involved and keeping the same people involved throughout was the best approach. Sixty-two respondents suggested that a combination of direct communication (e.g. through face to face or online meetings) and indirect communication (e.g. emails) was the ideal method of interaction with public partners. All six UK Standards for Public Involvement were considered important to consider within rapid evidence syntheses by at least 84.09% of participants. Most free text responses related to the need to ensure inclusive opportunities for people to be involved in rapid evidence syntheses (*N* = 27) and for clear communications (*N* = 26). Full demographic information and visuals of responses to all survey questions can be found in Appendix 9.

Fifteen people attended workshop one and 16 attended workshop two. In general, most participants across both workshops were aged between 30 and 49 years old, were of White ethnicity, and were based within the UK. In workshop one, participants indicated that the best ways to find patients and the public to be involved in rapid evidence syntheses were to: post the opportunity to be involved on a website dedicated to patient and public involvement in research, such as VOICE or NIHR Be Part of Research (*N* = 13; 93%); approaching groups with an interest in the condition being researched, such as charities and voluntary organisations (*N* = 12; 86%); and asking patients and members of the public that the researchers have already worked with previously to be involved (*N* = 8; 57%). Workshop one participants also indicated that the protocol and methods development stage (*N* = 12; 86%), the data synthesis stage (*N* = 9; 64%), and the write-up of the rapid evidence syntheses were the most important stages of rapid evidence syntheses to embed PPI. In workshop two, the initial draft of the framework was presented and participants asked to reflect on what was missing, what could be improved and what might facilitate implementation of the framework in future. From the options provided, the RIPPLES acronym was chosen by 11 of 16 participants in workshop two. Full details surrounding demographics and the results of the Zoom polls from both workshops are found in Appendix 10.

### The RIPPLES framework for PPI in rapid evidence synthesis

**Fig. 3:**
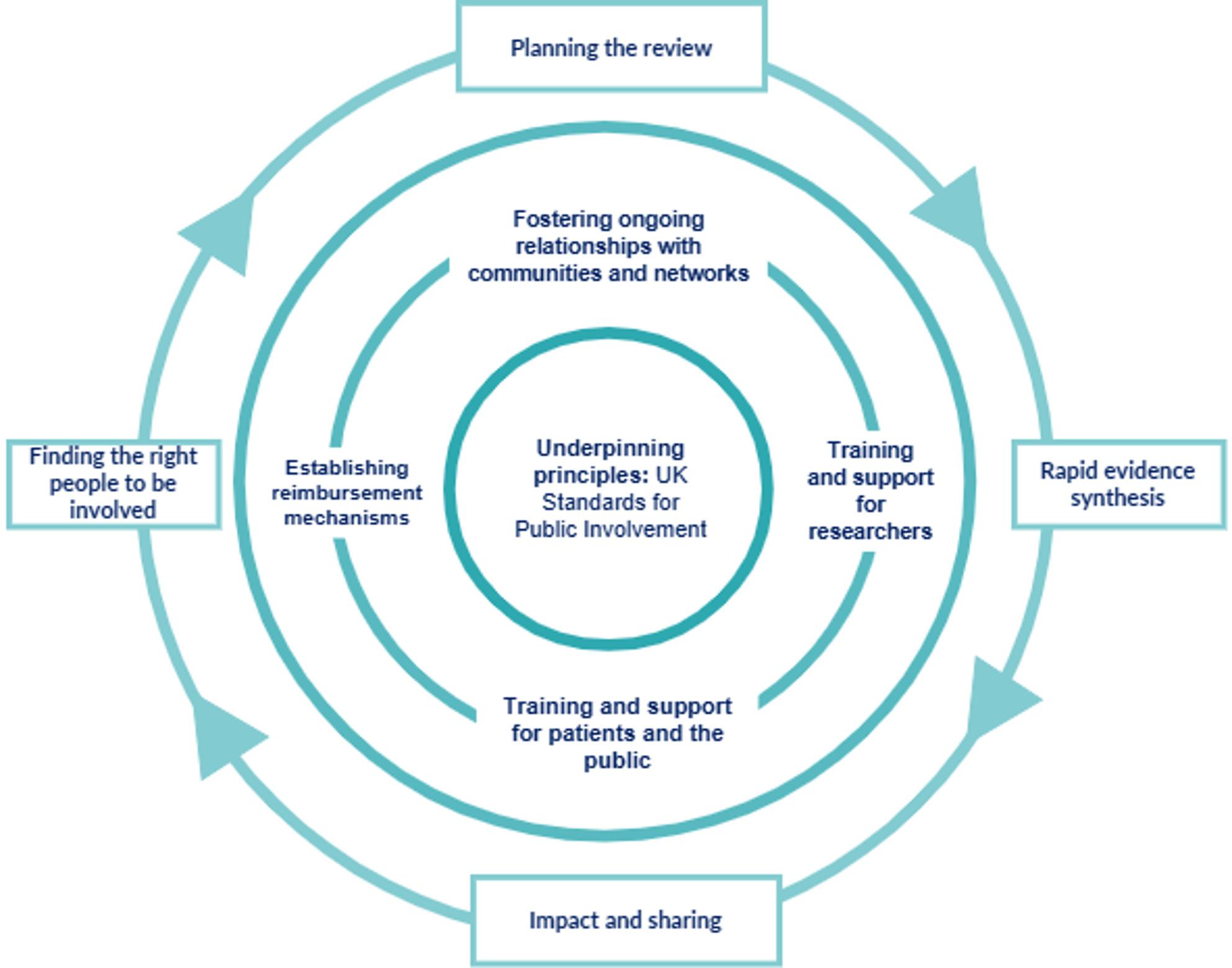
Visual overview of the RIPPLES framework. Abbreviations: UK = United Kingdom

A visual overview of the RIPPLES framework is shown in Fig. 3. RIPPLES is composed of three layers: core underlying principles at the centre; building continued approaches to patient, public and community involvement; and steps for embedding PPI into individual rapid evidence syntheses. The framework is accompanied by a detailed guidance document describing each of its components in more depth and a worksheet for researchers to effectively plan PPI in rapid evidence syntheses; these can be found on the NIHR Innovation Observatory website [16]. The remainder of this article provides a higher-level overview of each of the three layers of RIPPLES.

### Underlying principles: the UK standards for public involvement

The RIPPLES framework is underpinned by the six UK Standards for Public Involvement: communication; inclusive opportunities; working together; governance; impact; and support and learning [4]. The six principles each contain guiding questions to improve approaches towards involvement over time [17]. These principles should be interwoven throughout the other components of the RIPPLES framework and act as a foundation for good practice in PPI for rapid evidence synthesis.

### Continual approaches to patient, public and community engagement

The benefits of developing relationships with patients, the public and communities have been documented within the current literature surrounding PPI [18, 19]. The middle layer of the RIPPLES framework continues to reinforce these benefits, both for researchers and for wider communities. RIPPLES advocates for developing sustained relationships beyond individual rapid evidence synthesis projects with members of the public, community groups, networks, charities, voluntary sector organisations and faith groups to establishing inclusive working relationships with a diverse group of people. Reflecting on guidance such as NIHR INCLUDE and the co-produced Checklist for Inclusive Community involvement in health research (CHICO) can help provide guidance on how to approach these activities [20, 21]. 

Feeding into these continuing approaches to involvement, RIPPLES also advocates for training for both researchers and members of the public about PPI. This is particularly warranted as it has been noted that researchers often feel poorly equipped to undertake PPI [22]. To ameliorate this, the RIPPLES guidance document signposts to training that has already been developed for both PPI and researchers, including: the NIHR’s Learning for Involvement webpages; [23] the NIHR’s Public Information Pack; [24] and a “bite-size learning” module about systematic reviews for PPI developed by Cochrane and Evidence Synthesis Ireland [25]. RIPPLES also acknowledges that a wealth of expertise can also be found internally within institutions. Researchers can draw on the knowledge of colleagues to both enhance their networks and gain awareness of internal and external training opportunities.

The final aim of the middle layer of RIPPLES is to ensure there are mechanisms in place within institutions to adequately recognise or renumerate PPI for their time being involved in rapid evidence syntheses. Flexibility in how PPI are remunerated and recognised (e.g. via gift vouchers or bank transfers) is strongly recommended by the NIHR [12], though it has been acknowledged that the mechanisms for offering this flexibility often vary by institution.

We acknowledge this can be a time-consuming and resource intensive process. Indeed, structural and organisational barriers to meaningful PPI, such as budget constraints, limited funding for involvement, and lack of time and resource, have been previously documented [26]. The need for more structural support enabling researchers to undertake PPI has also been stated [22]. However, the overall aim of the middle layer of RIPPLES is to ensure that strong foundations are built to enable more efficient and meaningful facilitation of PPI within individual rapid evidence syntheses.

### PPI within rapid evidence synthesis

The outer layer of the RIPPLES framework focuses on how PPI can be embedded within the steps of rapid evidence syntheses, building on the foundations of the inner layers. The guidance document and worksheet assist researchers in planning PPI for individual rapid evidence syntheses, from finding the right people to be involved in each review to sharing the results and impact of the PPI. The framework and guidance document emphasise involvement in the planning, interpretation and dissemination stages of rapid evidence syntheses, as these were the parts of the review process emphasised as most crucial for PPI input by participants at workshop one. By finding the “right” people to be involved in rapid evidence syntheses, we mean identifying a range of people to represent the varied views and experiences of a topic as much as possible. Furthermore, the framework also includes a focus on mutual expectation-setting and transparently discussing what can be achieved within the time and resource considerations of each project.

Furthermore, in line with the UK Standards for Public Involvement, the RIPPLES guidance document also places an emphasis on communicating the impact of PPI within rapid evidence synthesis both to those who were involved in the process and to the wider research community. Providing opportunities for patients and members of the public to researchers is also essential in aiding understanding of how practice could potentially be improved in future projects. Frameworks such as the Public Involvement in Research Impact Toolkit (PIRIT) and the Public Involvement Impact Assessment Framework (PiiAF) can be useful tools for researchers in documenting the impact PPI has had on an individual project [27, 28]. Reporting of PPI within rapid evidence syntheses can also be enhanced and made more transparent by using the Guidance for Reporting Involvement of Patients and the Public 2 (GRIPP2) checklist [13]. 

Crucially, RIPPLES includes specific guidance surrounding how to embed PPI into reviews commissioned by interest-holders. Rapid evidence syntheses are often commissioned by interest-holders including healthcare organisations, government agencies and policymakers to inform time-sensitive decision-making [7]. As such, this means the scope and eligibility criteria of the review can often be pre-defined to fit a specific question. As this can preclude patients and members of the public being involved in defining the scope of the work, RIPPLES advocates for discussing how interest-holders have embedded PPI into the shaping of their research questions. The guidance document and worksheet take researchers through these questions and how this information can inform the wider rapid evidence synthesis.

At workshop 2, attendees noted that PPI is not linear and can be a cyclical process. Thus, while there are defined steps within the RIPPLES guidance, the arrows and feedback loop represent how the impact of PPI and shared learning can continually feed into future rapid evidence syntheses.

## Discussion

The RIPPLES framework is a practical guide for researchers to enable them to embed PPI into rapid evidence syntheses. It is built on established principles of best practice within PPI, including the UK Standards for Public Involvement and NIHR guidance surrounding inclusive involvement and engagement [4, 29], but has also been co-developed alongside researchers, members of the public, and PPI professionals to ensure it is responsive to their needs. It advocates for an approach that encourages the fostering of inclusive community partnerships while also being flexible to the demands of rapid evidence syntheses and the practical considerations of the methodology.

Indeed, in a prior scoping review of frameworks for PPI, Greenhalgh et al. (2019) suggested that “one size fits all” approaches may be less useful than single, standalone models [30]. We were also aware of the vast wealth of knowledge and tools already available to researchers surrounding PPI practice more broadly. As such, RIPPLES is not a standalone point of reference for researchers. The framework and its associated resources have been designed to be driven by and flexible within the context in which rapid evidence syntheses are conducted (e.g. whether the review is proactively driven by researchers or commissioned by interest-holders). Furthermore, we focused on guiding researchers specifically through the process of embedding PPI into rapid evidence synthesis but signpost to good practice resources wherever possible so as not to cause “research waste.”

### Strengths and limitations of this work

The major strengths of the RIPPLES framework lie in the use of three different methodologies to underpin development of the guidance: a scoping review; an interest-holder survey; and two workshops with key interest-holders. The methods followed a proposed outline for framework development [11]. Implementing these components meant we were able to co-develop the framework to best meet the needs of those who would use it. The co-development of the framework is evident in the complete redesign of the framework visual that took place after workshop 2 in response to participants’ feedback and ideas. Furthermore, we consistently involved two members of the public in the development of the framework (DS and RH), including collaborating with them in designing and delivering the workshops, helping to interpret the information, and feeding into the shape of the resources that we produced. We ensured that there was a feedback loop with our public partners, ensuring that both DS and RH knew how our approach had changed or been informed by their involvement, thus indicating the impact they had throughout the project.

However, the development of the framework is not without its limitations. The limitations of the scoping review have been discussed previously [31]. First, the survey was open to responses for two weeks, which may potentially have limited participation from a wider group of people. The use of snowball sampling may also have introduced selection bias into the survey [32]. Although our survey received 101 complete responses, most respondents were of white ethnicity (*N* = 62), with 26 responses from those of Black, African or Caribbean ethnicity and four of Asian ethnicity. Similarly, most of the attendees at the workshops were of white ethnicity (*N* = 11 in workshop one and *N* = 10 in workshop two). The lack of ethnic diversity in the participants responding to the survey and workshops may mean that issues and approaches that matter most to these communities could potentially be missing from the framework. An evaluation of the use of RIPPLES with a diverse range of people would help ensure that these perspectives are included.

Finally, the RIPPLES framework and its associated materials have been developed within the UK, where PPI is more formally established as a part of health and social care research. PPI is noted to be less formally established in other areas globally, including parts of Europe and lower and middle-income countries [33, 34]. As such, the framework and its guidance may be less representative or applicable to other research contexts globally.

### Implications for practice and research

In their review, Greenhalgh et al. (2019) noted that frameworks for PPI were often rarely used by people beyond those who had created them [30]. Our intention is for the RIPPLES framework to be adopted and used by researchers working on rapid evidence syntheses, to help guide their PPI practices. Discussions during workshop 2 highlighted that RIPPLES would likely be adopted not only by researchers working on rapid evidence syntheses, but also by PPI professionals and funders within the UK. We encourage the use of RIPPLES alongside reporting guidelines such as the interim guidance from the Preferred Reporting Items for Systematic Reviews and Meta-Analyses extension for Rapid Reviews (PRISMA-RR) and GRIPP2 [13, 35]. 

However, workshop 2 also highlighted potential challenges to implementing RIPPLES more broadly. These included: potential difficulty in being able to involve a diversity of voices and perspectives, particularly at short notice; a lack of infrastructure to support researchers implementing the training and relationship building advocated by RIPPLES; and how broader attitudes to PPI within research, particularly by those who may see PPI as a “tick box exercise”, could prove a barrier to implementation. To be responsive to these potential challenges, the RIPPLES framework will need to undergo evaluation and continual development. We intend for it to be a living resource that is refined and added to as we learn more about its adoption and implementation. We intend to design a structured evaluation plan and engage with people who have used RIPPLES. Through this evaluation process, we aim to identify: areas where the framework is working well; areas for refinement; and the need to develop further resources. Furthermore, evaluating RIPPLES will provide the opportunity to produce case studies on how the framework has been used in practice and its impact alongside those who have used it. This was a resource suggested by attendees at our workshops and may help demonstrate the impact of both RIPPLES and PPI more broadly within rapid evidence syntheses.

Finally, members of the public who were involved in our workshops highlighted that the current RIPPLES framework is aimed towards researchers. Although our initial aim for RIPPLES was to develop a framework that researchers, PPI professionals and policymakers could adopt, ensuring that members of the public can also understand and engage with the materials and its core concepts is imperative to ensuring its success. As part of our continuing efforts to develop the framework, we aim to co-develop public-facing materials about RIPPLES. Based on discussions within the workshops, this may include accessible, engaging and relevant resources such as: videos; infographics; and leaflets in plain language.

## Conclusions

RIPPLES is the first framework to offer specific guidance to researchers on how to involve patients and members of the public in rapid evidence syntheses. Its structure builds on the UK Standards for Public Involvement and advocates for relationship-building and training with researchers and communities, as well as offering detailed guidance on how to meaningfully embed PPI in this work. We will continue to develop RIPPLES and evaluate its use to ensure that it is fit for purpose and implementable in future.

## Supplementary Information

Below is the link to the electronic supplementary material.

Supplementary Material 1

## Data Availability

No datasets were generated or analysed during the current study.

## Abbreviations

ACTIVE: Authors and Consumers Together Impacting on eVidencE
CHICO: Checklist for Inclusive Community involvement in health research
GRIPP2: Guidance for Reporting Involvement of Patients and the Public
NIHR: National Institute for Health and Care Research
PiiAF: Public Involvement Impact Assessment Framework
PIRIT: Public Involvement in Research Impact Toolkit
PPI: patient and public involvement
RIPPLES: Rapid Involvement of Patients and the PubLic in Evidence Synthesis
UK: United Kingdom
