# Supplementary material for: Development of the RIPPLES framework for patient and public involvement in rapid evidence syntheses
Source: Res Involv Engagem. 2026 Apr 15;12:68. doi: 10.1186/s40900-026-00878-5 (PMC13192043; doi:10.1186/s40900-026-00878-5)

**Appendix 1: GRIPP-2 short form reporting checklist**

| **Section and topic** | **Item** | **Reported on page No** |
| --- | --- | --- |
| 1: Aim | Report the aim of PPI in the study | 4 |
| 2: Methods | Provide a clear description of the methods used for PPI in the study | 4-5 |
| 3: Study results | Outcomes—Report the results of PPI in the study, including both positive and negative outcomes | 4-5, 11 |
| 4: Discussion and conclusions | Outcomes—Comment on the extent to which PPI influenced the study overall. Describe positive and negative effects | 4-5, 11 |
| 5: Reflections/critical perspective | Comment critically on the study, reflecting on the things that went well and those that did not, so others can learn from this experience | 11 |

**Appendix 2: Survey design**

**Help us to create a framework that researchers can use to involve patients and the public in rapid evidence syntheses**

**Who are we looking for?**

We are looking for anyone with an interest in patient and public involvement in evidence synthesis research to complete this survey. You must be 18 or older to express your interest.

**What does this project involve?**

We are developing a framework to help researchers involve patients and the public in rapid evidence syntheses. A framework can be thought of simply as a clear and easy to follow process that anyone could follow to ensure they get the desired outcome, which in this case is good, effective and meaningful public involvement. Evidence synthesis involves combining information from multiple different studies and sources on the same topic to understand their findings. By using evidence syntheses, policymakers, clinicians, the public and researchers can make more informed decisions about healthcare. Sometimes these evidence syntheses are done in just a few weeks.

The National Institute for Health and Care Research (NIHR) advocates for involving patients and the public in all health research. However, we currently do not have any guidance on how to involve people meaningfully in evidence syntheses with very short timelines for completion. We want to create this framework to enable researchers doing rapid evidence syntheses involve patients and the public in their work. To make it as fit for purpose as possible, we want to create this framework with the input of the public, researchers, policymakers and clinicians.

**What will taking part in the survey involve?**

In this survey, we will ask you to provide some demographic information (such as your age group and your ethnicity) to ensure that we are capturing as broad a range of perspectives as possible, but you will also be given the option not to disclose this information. You will then be asked a series of multiple-choice and open-ended questions asking for your opinion on how patient and public involvement in rapid evidence syntheses should be done. For each question, you will be asked to consider how a research team could conduct patient and public involvement for a project lasting two months.

We anticipate that the survey will take 10 to 15 minutes of your time.

Following our survey, we will also be hosting two online workshops in October and November 2024 to help us build on the results of this survey and develop this framework. At the end of this survey, you will be asked if you would be interested in attending these workshops. You will be asked to provide your name and email address if you express an interest, so we can contact you in future. We only have limited places in each workshop and we are aiming to include a mix of perspectives in the workshops. Unfortunately, this means we cannot guarantee everyone who expresses an interest a place but we will contact everyone who has expressed an interest to let them know whether or not they have been selected.

This study was approved by the Faculty of Medical Sciences Research Ethics Committee, part of Newcastle University’s Research Ethics Committee. This Committee includes members who are internal to the Faculty. This study was reviewed by members of the Committee, who must provide impartial advice and avoid significant conflicts of interests.

**What data will we collect and store?**

We will summarise some information you provide in this survey (e.g. age group and ethnicity) within our report and any subsequent publications, so that we can provide some general information about the group of contributors who completed the survey. We will also ask you for your name and email address so we can contact you, if you express an interest in being a part of a workshop.

We will store anonymised data from the survey for up to two years for research purposes. If you express an interest in being a part of the workshops, we will securely store email addresses for up to 12 weeks following completion of the study to inform participants of its outcomes.

We will store all the data collected in this survey securely on an encrypted Newcastle University platform and will not share the data with anyone beyond the research team.

Newcastle University will act as the data controller for this study, ensuring your information is used appropriately and in the public interest. You can find out more about this here or by contacting Newcastle University's Data Protection Officer (Maureen Wilkinson, rec-man@ncl.ac.uk).

**Can I stop taking part?**

Your participation in this survey is voluntary. If you do decide to take part, you can stop taking part in this survey at any point for any reason. You can also let us know if you want to withdraw your interest by emailing us, but please note that this may not be possible once data analysis has begun.

**Who do I contact if I have a concern about the research, or I wish to complain?**

If you have a concern or any queries about any aspect of this study, please contact: insights@io.nihr.ac.uk

**End of Block**

Q1 Are you 18 years old or over?

- Yes (1)
- No (2)

**End of Block**

Q2 **To take part in this survey, please provide your consent to participate.**

I confirm that I have read and understood the previous information regarding the study and agree to participate in the survey.

- Yes (1)
- No (2)

**End of Block**

Q3 How would you best describe yourself?

- Clinician (1)
- Member of the public (2)
- Researcher (3)
- Policymaker (4)
- Prefer not to say (5)
- Other (please describe) (6) __________________________________________________

Q4 Where are you currently living?

- In the UK (1)
- In Europe (2)
- Outside of Europe (3)
- Prefer not to say (4)

Q5 What is your age range?

- 18 to 29 (1)
- 30 to 49 (2)
- 50 to 64 (3)
- 65 and over (4)
- Prefer not to say (5)

Q6 What is your ethnic group?

- White (1)
- Asian (2)
- Black/ African/ Caribbean (3)
- Chinese (4)
- Arab (5)
- Other ethnic group (6)
- Prefer not to say (7)

**End of Block**

Q7 Have you ever been involved in a rapid evidence synthesis project, either as a researcher, clinician, member of the public or in another role?

- Yes (1)
- No (2)
- Not sure (3)

**End of Block**

Q8 If you were involved in an evidence synthesis project lasting two months, how would you propose recruiting people to be involved in the research?

- Having an open call for people to be involved and keeping the same people involved throughout the project (1)
- Having an open call for people to be involved and taking a flexible approach to who is involved throughout the project (i.e. not having the same people involved at all stages of the project) (2)
- Inviting specific individuals to be involved in the research (3)
- Inviting a specific, existing group to be involved in the research (4)
- None of these (5)

Q9 If you were involved in an evidence synthesis project lasting two months, how would you propose that the people running the research and those involved interact with each other?

- Direct interaction (e.g. though face to face or online meetings) (1)
- No direct interaction (e.g. only communicating through email) (2)
- A combination of these (3)
- None of these (4)

**End of Block**

Q10 If you were involved in an evidence synthesis project lasting two months, how important would the following principles of patient and public involvement and engagement be to you?

|  | Important (1) | Not important (2) |
| --- | --- | --- |
| Involving a range of people and groups, as informed by community and research needs (1) |  |  |
| Working together in a way that values all contributions, and that builds and sustains mutually respectful and productive relationships (2) |  |  |
| Offering and promoting support and learning opportunities that build confidence and skills for public involvement in research (3) |  |  |
| Involving the public in research management, regulation, leadership and decision making (4) |  |  |
| Using plain language for well-timed and relevant communications as part of involvement plans and activities (5) |  |  |
| Seeking improvement by identifying and sharing the difference public involvement makes to research (6) |  |  |

**End of Block**

Q11 Please use the space below to tell us anything else you think we may have missed or should consider about involving patients and the public in rapid evidence syntheses.

________________________________________________________________

**End of Block**

Q12 Thank you for completing this survey. We will be hosting two online workshops on Wednesday 9th October and Wednesday 20th November 2024 to help co-create the framework and then refine our draft. We only have limited places in each workshop and we are aiming to include a mix of perspectives. Unfortunately, this means we cannot guarantee everyone who expresses an interest a place but we will contact everyone who has expressed an interest as soon as we can to let them know whether or not they have been selected. Would you be interested in attending these online workshops? Each workshop will last around 2 hours.

- Yes (1)
- No (2)

**End of Block**

Q13 Thank you for expressing an interest in joining the workshops. So that we can contact you with further details if you are selected to attend, please provide your name and email address.

- Name (1) __________________________________________________
- Email (2) __________________________________________________

**End of Block**

**Appendix 3: Consent form for workshops**

**Help us to create a framework that researchers can use to involve patients and the public in rapid evidence syntheses**

**Who are we looking for?**

You have previously indicated that you would be interested in joining workshops to help us create a framework that researchers can use to involve patients and the public in rapid evidence syntheses.

Before deciding to take part, it is important you understand why the research is being done and what it will involve. Please read the following carefully and discuss it with others if you wish.

If anything is not clear or you would like more information, please email: insights@io.nihr.ac.uk

**Why are we doing these workshops?**

We are developing a framework to help researchers involve patients and the public in rapid evidence syntheses. A framework can be thought of simply as a clear and easy to follow process that anyone could follow to ensure they get the desired outcome, which in this case is good, effective and meaningful public involvement. Evidence synthesis involves combining information from multiple different studies and sources on the same topic to understand their findings as a whole. By using evidence syntheses, policymakers, clinicians, the public and researchers can make more informed decisions about healthcare. Sometimes these evidence syntheses are done in just a few weeks.

The National Institute for Health and Care Research (NIHR) advocates for involving patients and the public in all health research. However, we currently do not have any guidance on how to involve people meaningfully in evidence syntheses with very short timelines for completion. We want to create this framework to enable researchers doing rapid evidence syntheses involve patients and the public in their work. To make it as fit for purpose as possible, we want to create this framework with the input of the public, researchers, policymakers and clinicians.

**What will taking part in a workshop involve?**

We will be hosting two, two-hour online workshops on 12th November 2024 (10.00am to 12.00pm) and 22nd January 2025 (1.00pm to 3.00pm) to help us develop this framework. In the first workshop, we will be presenting findings from research we have done to find out more about what features current frameworks for involving people in research contain. We will then discuss these findings and do activities to find out which of these features people think should be embedded into this new framework and those that might be less important.

In the second workshop, we will present a draft version of the framework, which will have been developed based on the findings from the first meeting. We will ask participants to highlight any areas for improvement and identify any potentially missing or irrelevant features. We will then use these findings to finalise our framework.

For your time, you will receive a £50 digital gift voucher for each of the workshops you attend. [NB: Included for consent forms to members of the public only]

This study was approved by the Faculty of Medical Sciences Research Ethics Committee, part of Newcastle University’s Research Ethics Committee. This Committee includes members who are internal to the Faculty. This study was reviewed by members of the Committee, who must provide impartial advice and avoid significant conflicts of interests.

**What data will we collect and store?**

We will ask you to provide your name as an indication of consent to take part and your e-mail to allow us to contact you about outcomes of the study. We will keep this data separate to any other data collected. We will summarise some of the information you previously provided in the survey (e.g. age group and ethnicity) within our report and any subsequent publications. We will ask you for your name and email address in this consent form so we can contact you but we will not publish any information that will personally identify you. The online workshops will be audio and video recorded using a Zoom recording function to allow for accurate capture of discussions.

Data recordings and transcripts from the workshops will be stored securely using a Newcastle University encrypted platform which only the researchers involved in the project will have access to. We may also use online tools, such as Mentimeter or Padlet, to help facilitate the workshops but will ensure that any data gathered on these tools is anonymous and not identifiable. We will only disclose information you provide if there is a legal requirement to do so (for example, under statute or a court order) and/or we have an overriding duty to the public (for example, the information concerns the commission of a criminal offence or relates to life-threatening circumstances). We will keep anonymised data for up to two years for research purposes and names and email addresses for up to 12 weeks following completion of the study to inform participants of its outcomes.

Newcastle University will act as the data controller for this study, ensuring your information is used appropriately and in the public interest. You can find out more about this here or by contacting Newcastle University's Data Protection Officer (Maureen Wilkinson, rec-man@ncl.ac.uk).

**Are there any risks in taking part?**

Although we will not be asking you to disclose any personal experiences during the workshops, if you experience distress during the focus group, you can pause or stop your participation as needed, or you may withdraw from the focus group.

**Can I stop taking part?**

If you do decide to take part, you can stop at any point without a reason just by letting the researcher know. If you stop after the recording of the workshops has been transcribed or anonymised, your consent form and email address will be deleted but we will be unable to identify your interview data for removal.

**Who do I contact if I have a concern about the research, or I wish to complain?**

If you have a concern or any queries about any aspect of this study, please contact insights@io.nihr.ac.uk

**End of Block**

Q1 Please indicate which of the workshops you are able to attend.

- 12th November 2024 - 10.00am to 12.00pm (1)
- 22nd January 2025 - 1.00pm to 3.00pm (2)
- Both (3)
- Neither of these (4)

**End of Block**

Q2 Please complete the following consent form while considering the information you have read above and/or subsequently discussed.

I confirm that I have read the information sheet provided, had the opportunity to consider the information, had the opportunity to ask questions and have had any questions answered satisfactorily.

- Yes (1)
- No (2)

Q3 I understand my participation is voluntary and that I can stop at any time. I understand that if I stop, any data I have provided up to that point (as far as is plausible) will be deleted.

- Yes (1)
- No (2)

Q4 I understand how my personal information will be used for this study.

- Yes (1)
- No (2)

Q5 I understand that I will be recorded, that this recording will be stored anonymously on password-protected software, used for research purposes only, then destroyed after completion of the transcription.

- Yes (1)
- No (2)

Q6 I understand that personal details, such as my name and email address, will not be revealed to anyone outside of the project.

- Yes (1)
- No (2)

Q7 I understand and agree my data may be published as a journal article and may appear in other materials used to share the findings of the project.

- Yes (1)
- No (2)

Q8 I am happy for my e-mail address to be stored for up to 12 weeks following completion of the study, so I can receive a gift voucher upon study completion and receive information about the outcomes of the study. [NB: Included for consent forms to members of the public only]

- Yes (1)
- No (2)

**End of Block**

Q9 Please confirm that you agree (consent) to take part in this research project by signing below.

- Name (1) __________________________________________________
- Email address (2) __________________________________________________
- Date (3) __________________________________________________

**End of Block**

**Appendix 4: Agenda for workshop one**

| **Name** | Workshop 1: Co-designing aspects of the PPIE framework for rapid evidence synthesis |
| --- | --- |
| **Facilitators** | Madeleine Still, Eugenie Evelynne Johnson, Sean Gill, Daisy Trenchard, Debbie Smith, Megan Fairweather, Kate Lanyi |
| **Date** | 12 November 2024; 10.00-12.00 |
| **Length** | 2 hours (120 mins) |

| **Activity** | **Minutes** | **Roles** |
| --- | --- | --- |
| **Welcome**  Welcome  Brief introduction to research team  Housekeeping  Icebreaker: name, where the person is calling from | 15 | EEJ |
| **Introduction**  What is rapid evidence synthesis and why we are doing this work  Specific aims of the session | 10 | EEJ |
| **Q&A**  Short Q&A to answer any questions about the project before workshop begins  Alert people that recording of the session will begin at this point | 10 | EEJ |
| **BEGIN RECORDING** | | |
| **Whole Group Activity 1: Recruitment – Zoom Poll**  Run through the scenario and the instructions for the task on PowerPoint  Allow people to vote on the poll  Note that the poll window may need to be maximised to see all the options available  Give technical support and assistance as required  Summarise the overall findings of the activity and explain that we will now go into smaller breakout rooms to discuss the activity in more detail  Facilitators to note most favoured options to take into breakout spaces | Explanation: 5  Activity: 5 | SG |
| **Small Group Activity 1 – Breakout Rooms**  **Facilitators: remember to record the breakroom room**  Breakout rooms according to role (5/6 people per room)  Following on from whole group activity 1  Summarise the key findings from the main activity again  **Prompts for discussion**:   - Most people picked X three responses; do you generally agree that these would be the preferred ways to find patients and members of the public for a very short project like this? - Why did you think this was the quickest way to find people? - Would there be anything more that researchers would need to consider when looking for patients and members of the public for these projects?   - e.g. Diversity and inclusion?   - e.g. Time and capacity?   - e.g. How much people already know about evidence synthesis or how much they would need training? - What would be the best way to try and address these issues when researchers are looking for people to be involved in these really short projects? | 20 | Room 1 (public): SG, DS  Room 2 (researchers + others): MS, DT  Room 3 (researchers + others): EEJ, MF  Main room (tech support): KL |
| **Feedback from Activity 1**  Facilitators to summarise overarching ideas from each breakout room | 5 | Facilitate: EEJ  Feedback:  EEJ, SG, MS |
| **Comfort break** | 10 | ALL |
| **Whole Group Activity 2: PPI in stages of the evidence synthesis process – Zoom Poll**  Run through the scenario and the instructions for the task  Allow people to vote on the poll  Note that the poll window may need to be maximised to see all the options available  Give technical support and assistance as required  Summarise the overall findings of the activity and explain that we will now go into smaller breakout rooms to discuss the activity in more detail  Facilitators to note most favoured options to take into breakout spaces | Explanation: 5  Activity: 5 | DT |
| **Small Group Activity 2 – Breakout Rooms**  **Facilitators: remember to record the breakroom room**  Breakout rooms according to role (5/6 people per room)  Following on from whole group activity 2  Summarise the key findings from the main activity again  **Prompts for discussion**:   - Most people picked X 3 responses; do you generally agree that these are the most important parts of the rapid evidence synthesis process for patients and members of the public to be involved in? - Why did you think these were the most important parts of the process to be involved in? - How can we best support involvement in these parts of the process?   - For both researchers and members of the public (what they both need to facilitate this) | 20 | Room 1 (public): SG, DS  Room 2 (researchers + others): MS, DT  Room 3 (researchers + others): EEJ, MF  Main room (tech support): KL |
| **Feedback from Small Group Activity 2**  - Facilitators to summarise overarching ideas from each breakout room | 5 | Facilitate: EEJ  Feedback:  EEJ, SG, MS |
| **Outline for next steps and workshop 2**  - What we will do with the information we have collated  - How it might feed into the framework  - How we will present what we’ve found and an initial framework draft at the workshop in January and ask for feedback and refinements | 10 | EEJ |
| **Close** | | |

**Appendix 5: Vignettes used within workshop one**


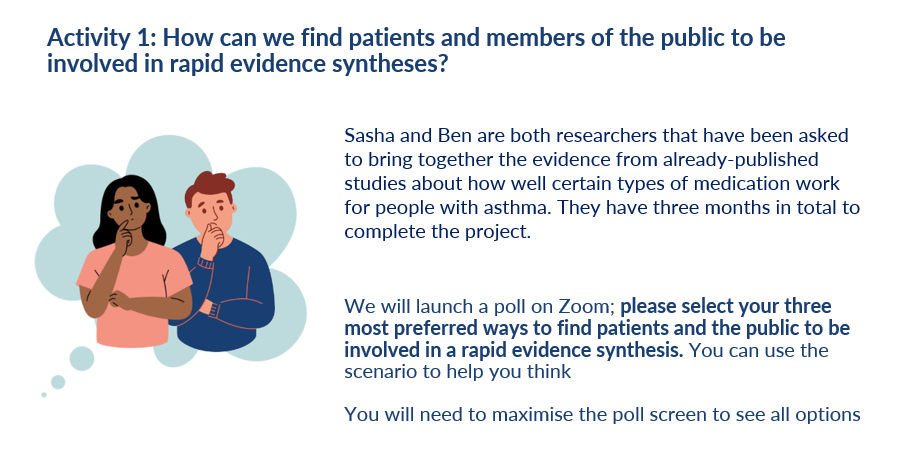


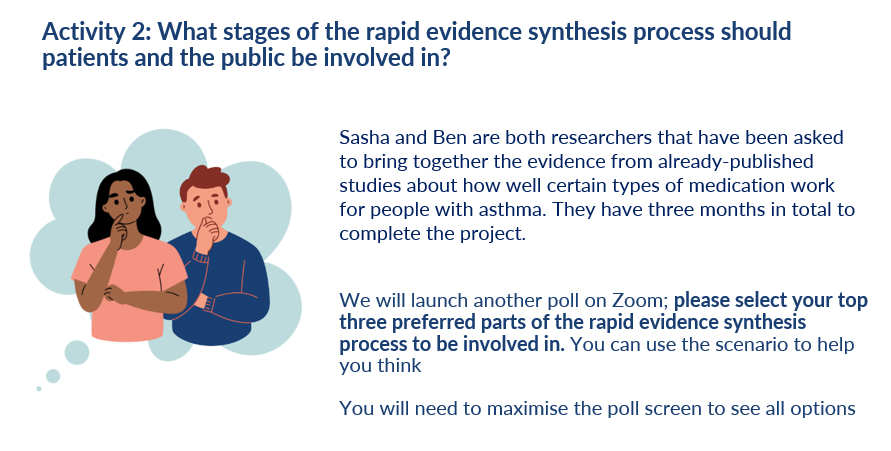


**Appendix 6: Coding framework for workshop one analysis**

| **Domain** | **Concept** |
| --- | --- |
| Recruitment | - Open recruitment - Closed recruitment - Inclusivity - Challenges |
| Stages of the rapid evidence synthesis process | - Protocol/scoping - Data synthesis - Writing the manuscript/ report - Other stages of the process |
| Inductive themes (added during coding process) | - Ongoing updates for PPI - Feedback - Reimbursement - Need for training - Engagement outside of projects - Community groups and organisations - Expectation setting - Feasibility - How to host meetings - Considerations for commissioned reviews - Patient/public knowledge - How to communicate - Value of involvement - Bias - Choices - Recognition for participation - Time |
| Abbreviations: PPI = patient and public involvement | |

**Appendix 7: Initial draft of the RIPPLES framework following workshop one**


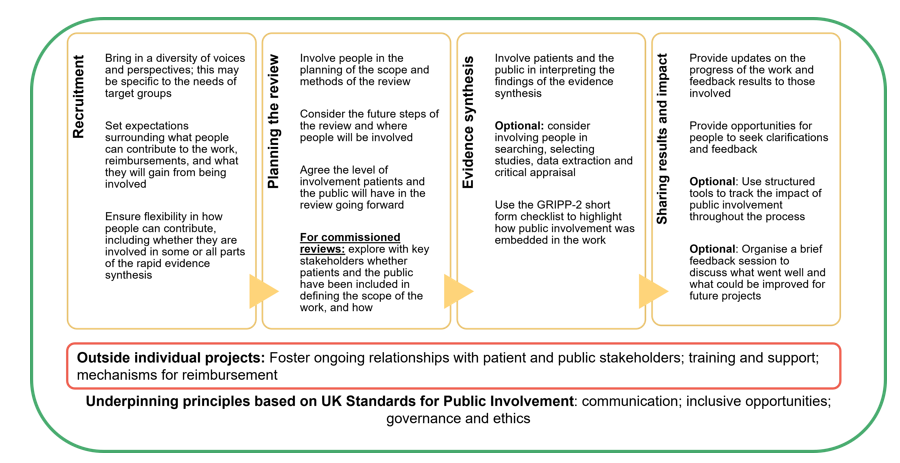


**Appendix 8: Agenda for workshop two**

| **Name** | Workshop 2: Presenting and refining the PPIE framework for rapid evidence synthesis |
| --- | --- |
| **Facilitators** | Madeleine Still, Eugenie Evelynne Johnson, Sean Gill, Daisy Trenchard, Jane McDermott, Kate Lanyi, Georgie Wilkins, Becky Harmston, Kirsti Brock |
| **Date** | 22 January 2025; 13.00-15.00 |
| **Length** | 2 hours (120 mins) |

| **Activity** | **Minutes** | **Roles** |
| --- | --- | --- |
| **Welcome**  Welcome  Brief introduction to research team  Housekeeping  Remind participants that the workshop will be recorded and note confidentiality | 15 | EEJ |
| **Introduction**  Short reminder about the project and what we are hoping to achieve with the framework  Overview of how we have come to workshop 2: steps to this point, how we incorporated workshop 1 into what we have done  Specific aims of workshop 2  Alert people that recording of the session will begin at this point | 10 | EEJ |
| **Begin recording here** | | |
| **Introducing the framework so far (note: participants will have been sent the framework draft a few days in advance to consider, if they wish)**   - Talk though each part of the framework - Note that the framework is underpinned by the UK Standards for Public Involvement - Note that in any publications or if we post the framework online that we will also include examples, further details on how to implement the guidance and further resources wherever possible - the overall framework here is just to give an overview of the major points included | 10 | 1: SG – Overview, underlying principles, recruitment, planning the review  2: DT – Evidence synthesis, sharing and impact |
| **Introduction to breakout room 1**  Explain that we will now go into smaller breakout rooms to discuss if anything might be missing from the current draft of the framework, if anything needs to be clarified and if anything could make it more usable. | 5 | DT |
| **Small group activity 1 – Breakout rooms**  **Facilitators: remember to record the breakroom room**  Breakout rooms according to role (5/6 people per room)  **Prompts for discussion**   - What are your initial thoughts on what has been included in the framework? - Is there anything you think is missing? - Is there anything you think needs more clarity? - Is there anything that could be added or amended to make these sections of the framework more usable? | 20 | ALL (in breakout rooms) |
| **Feedback from Activity 1**  Facilitators to summarise overarching ideas from each breakout room | 5 | ALL |
| **Comfort break** | 5 | ALL |
| **Lead into breakout room session 2**  Explain that we will now go into smaller breakout rooms to discuss how we might implement the framework and what we could do to overcome any challenges. | 5 | MS |
| **Small group activity 2 – Breakout rooms (Implementation)**  **Facilitators: remember to record the breakroom room**  Breakout rooms according to role (5/6 people per room)  **Prompts for discussion (N.B this may look slightly different for public room)**   - How easily/ what challenges might you experience implementing this framework - Are there examples from past work where features/elements of this framework have been successful? - Are there any tools that could aid in the implementation of the framework that already exist or may be worth investigation in future? | 20 | ALL (in breakout rooms) |
| **Feedback from Activity 2**  Facilitators to summarise overarching ideas from each breakout room | 5 | ALL |
| **Whole group activity: Naming the framework (Zoom Poll)**   - Explain that we have narrowed down some options for a quick name for the framework using an acronym generator and the words “patient public involvement rapid evidence synthesis” - We would like the group to vote on which of the options they most prefer, which will become the short name for the framework going forward - Launch poll on Zoom - Feedback result   **Current options:**   - **RIPPLES** - Rapid Involvement of Patient and PubLic in Evidence Synthesis - **PIPPIN** - PatIent Public raPid evIdence syNthesis - **ENLIVEN** - patiENt pubLic InvolVement Evidence syNthesis - **ENRICH** - patiEnt iNvolvement Rapid evIdenCe syntHesis | 5 | EEJ |
| **Next steps and feedback**   - Explanation that the insights gained across the course of this workshop will be used to further refine the framework before it is finalised | 10 | EEJ |
| **Close** | | |

**Appendix 9: Coding framework for workshop two analysis**

| **Domain** | **Concept** |
| --- | --- |
| Deductive domains | - Missing elements from the framework - Extraneous elements from the framework - Points for clarification in framework draft - Look/design: positives - Look/design: improvements - Implementation challenges - Implementation facilitators - Resources the RIPPLES team could develop - Useful resources (external) |
| Inductive themes (added during coding process) | - Ideas for planning evaluation |
| Abbreviations: PPI = patient and public involvement | |

**Appendix 10: Demographic information and responses to online survey**

*Characteristics of survey respondents (N=101)*

*Characteristics of survey respondents grouped by age group, ethnic group and if they had participated in a rapid evidence synthesis previously (N=101)*


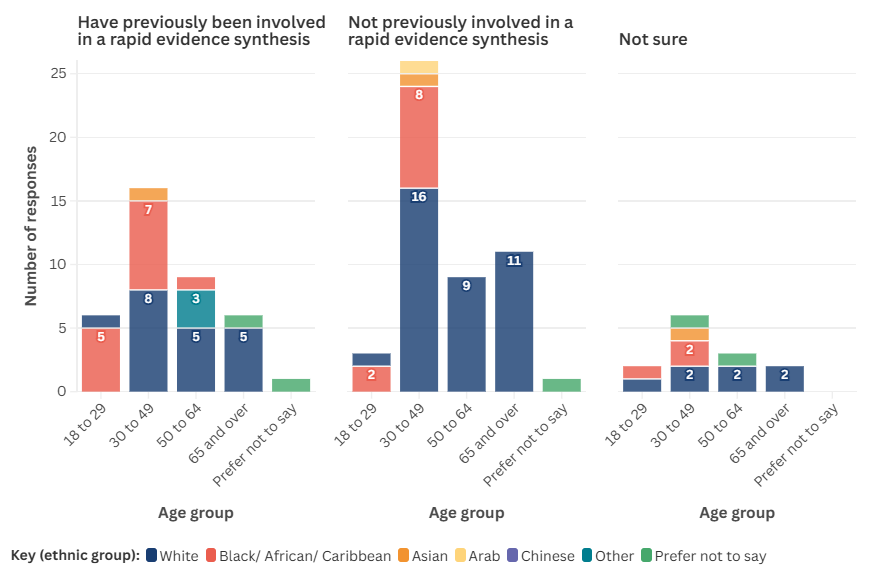


*Preferences for recruitment method according to survey respondents (N=101)*


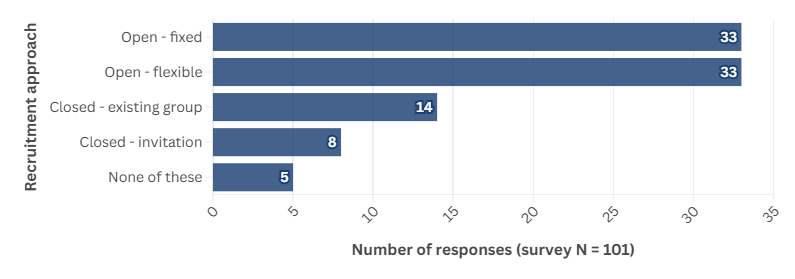


*Preferences for interaction method according to survey respondents (N=101)*


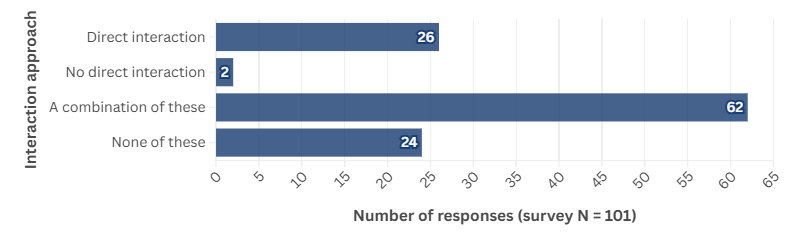


*Importance of the UK Standards for Public Involvement for survey respondents (N=101)*


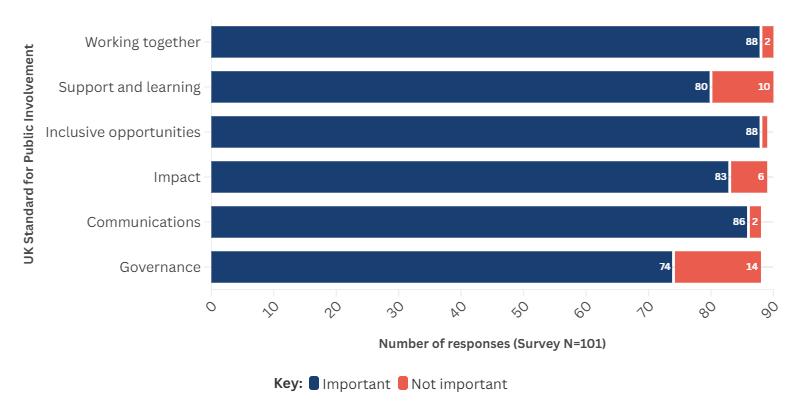


*Sunburst diagram showing themes from qualitative responses in the survey grouped by corresponding UK Standard for Public Involvement*


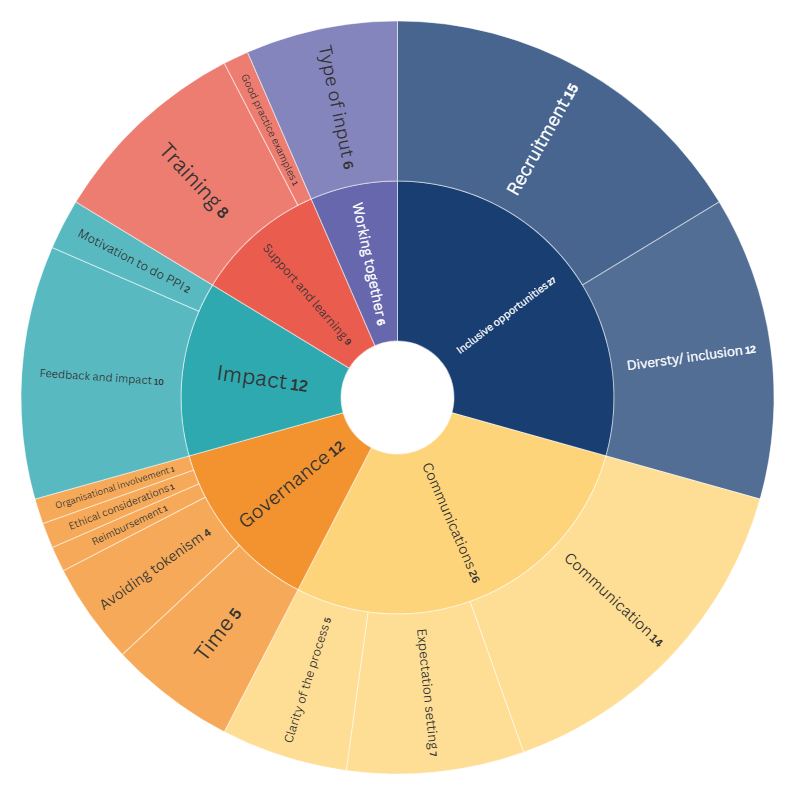


**Appendix 11: Demographic information for workshops and results of Zoom polls**

*Demographic information*

| **Characteristic** | **Workshop 1 (n=15)** | **Workshop 2 (n=16)** |
| --- | --- | --- |
| **Role** | | |
| Member of the public | 5 | 5 |
| Researcher | 6 | 7 |
| Policymaker | 1 | 0 |
| Clinician | 0 | 0 |
| Other | 3 | 4 |
| **Age group** | | |
| 18 to 29 | 1 | 1 |
| 30 to 49 | 8 | 11 |
| 50 to 64 | 5 | 3 |
| 65 and over | 1 | 0 |
| Prefer not to say | 0 | 1 |
| **Ethnic group** | | |
| White | 11 | 10 |
| Black/ African/ Caribbean | 2 | 1 |
| Asian | 0 | 1 |
| Arab | 1 | 1 |
| Chinese | 1 | 1 |
| Other | 0 | 1 |
| Prefer not to say | 0 | 1 |
| **Geographic location** | | |
| In the UK | 13 | 14 |
| In Europe | 2 | 1 |
| Outside of Europe | 0 | 1 |

*Workshop one: Results of poll asking attendees to consider the best ways to find people to be involved in rapid evidence syntheses*


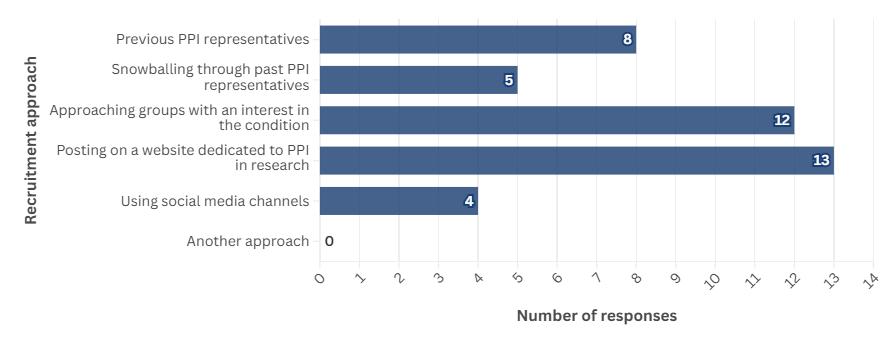


*Workshop one: Results of poll asking attendees to consider which of the stages of the rapid evidence synthesis process it would be most important to include PPI in*


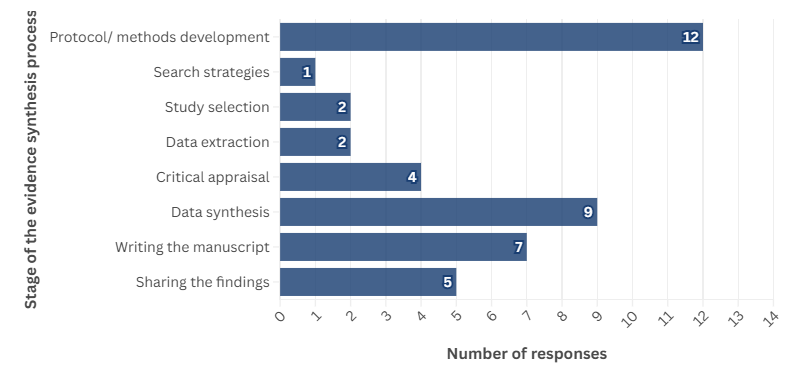


*Workshop two: Results of poll asking attendees to select a name for the framework*


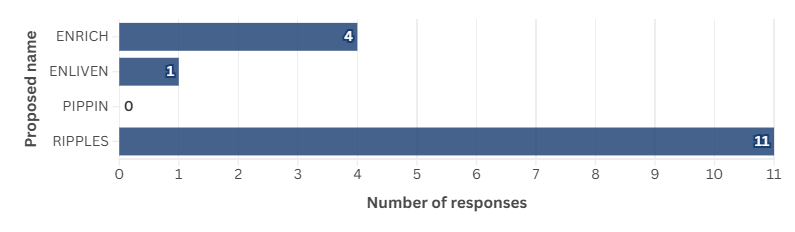

Supplement: Supplementary file 1 — Supplementary Material 1 [file 40900_2026_878_MOESM1_ESM.docx]
